# SUMOylation Represses *Nanog* Expression via Modulating Transcription Factors Oct4 and Sox2

**DOI:** 10.1371/journal.pone.0039606

**Published:** 2012-06-22

**Authors:** Yongyan Wu, Zekun Guo, Haibo Wu, Xiaohai Wang, Lixia Yang, Xiaoyan Shi, Juan Du, Bo Tang, Wenzhong Li, Liping Yang, Yong Zhang

**Affiliations:** 1 College of life Sciences, Northwest A&F University, Yangling, Shaanxi, China; 2 College of Veterinary Medicine, Northwest A&F University, Key Laboratory of Animal Biotechnology, Ministry of Agriculture, Yangling, Shaanxi, China; Kanazawa University, Japan

## Abstract

Nanog is a pivotal transcription factor in embryonic stem (ES) cells and is essential for maintaining the pluripotency and self-renewal of ES cells. SUMOylation has been proved to regulate several stem cell markers' function, such as Oct4 and Sox2. Nanog is strictly regulated by Oct4/Sox2 heterodimer. However, the direct effects of SUMOylation on *Nanog* expression remain unclear. In this study, we reported that SUMOylation repressed *Nanog* expression. Depletion of Sumo1 or its conjugating enzyme Ubc9 increased the expression of Nanog, while high SUMOylation reduced its expression. Interestingly, we found that SUMOylation of Oct4 and Sox2 regulated *Nanog* in an opposing manner. SUMOylation of Oct4 enhanced Nanog expression, while SUMOylated Sox2 inhibited its expression. Moreover, SUMOylation of Oct4 by Pias2 or Sox2 by Pias3 impaired the interaction between Oct4 and Sox2. Taken together, these results indicate that SUMOylation has a negative effect on Nanog expression and provides new insights into the mechanism of SUMO modification involved in ES cells regulation.

## Introduction

Derived from the inner cell mass (ICM) of the blastocyst, ES cells can proliferate indefinitely in vitro and differentiate into cells of all three germ layers. These unique properties make ES cells exceptionally valuable for cell replacement therapies, drug discovery and regenerative medicine [1], [2]. An intricate network of transcription factors has been found in undifferentiated ES cells for maintaining its features. And recent studies indicated that Nanog, a homeobox transcription factor, was involved in this network and played a critical role in regulating the cell fate of the pluripotent ES cells [3]. Nanog is expressed in ES cells and is thought to be a key factor in maintaining ES cells pluripotency. It functions together with other factors such as Oct4 and Sox2 to establish ESC identity [4]–[6]. In addition, Nanog is essential for early embryonic development, and is regarded as the gateway for somatic cells to reprogram into induced pluripotent cells [7].

The small ubiquitin-like modifier (SUMO) proteins are structurally similar to ubiquitin although they share less than 20% sequence identity. Like ubiquitylation, protein SUMOylation is regulated by a cascade of reactions involving SUMO-activating enzymes (SAE1/SAE2), conjugating enzymes (Ubc9) and multiple E3 ligases (e.g. PIAS1, PIAS2, PIAS3, PIAS4 (PIASy), RanBP2 and Pc2) that covalently attach SUMO to specific protein substrates. In addition, a number of de-SUMOylation enzymes (i.e. Ulp/SENPs) for rapid deconjugation are core components of this reversible post-translational modification [8].

In lower eukaryotes, a single SUMO gene is expressed (*Smt3* in *Saccharomyces cerevisiae*), whereas in vertebrates three paralogs designated as SUMO1–3 are ubiquitously expressed in all tissues, the human genome also encodes a gene for SUMO4 that appears to be uniquely expressed in the spleen, lymph nodes and kidney [9]. Ubc9 is the sole E2 enzyme for SUMOylation [10]. SUMO E3 ligases are the enzymes assumed to ensure substrate specificity, and most E3 ligases interact with both the SUMO-Ubc9 thioester and substrate to bring them in close proximity for SUMO transfer [11].

Covalent modification of proteins by small ubiquitin-like modifiers (SUMO) cause changes in the intracellular localization and stability of proteins, and alters their abilities to interact with other proteins and nucleic acids. In particularly, these modifications affect the functions of proteins involved in a wide range of cellular processes [8], [12]–[14], including macromolecular transport, the maintenance of nuclear structure, nucleic acid DNA metabolism and cell signaling.

The most well-known group of SUMO substrates is transcription factors, in which SUMOylation regulates transcriptional activity. Previous studies have revealed that SUMOylation can positively or negatively regulate the transcriptional activity of pluripotent factors such as Oct4 and Sox2, which play critical roles in the maintenance of ES cell pluripotency and promote reprogramming of fibroblasts [15]–[17], thereby linking SUMOylation with pluripotency. *In vivo*, the expression of Nanog is strictly regulated by the Oct4/Sox2 heterodimer and other transcription factors [18]. To further investigate the role of SUMOylation in the regulatory gene network of ES cells, we examined the effect of SUMOylation on *Nanog* expression. Our results showed that SUMOylation of transcription factors Sox2 and Oct4 regulates their transcriptional activity differentially and represses Nanog expression.

## Results

### SUMOylation represses Nanog expression

**Figure 1:**
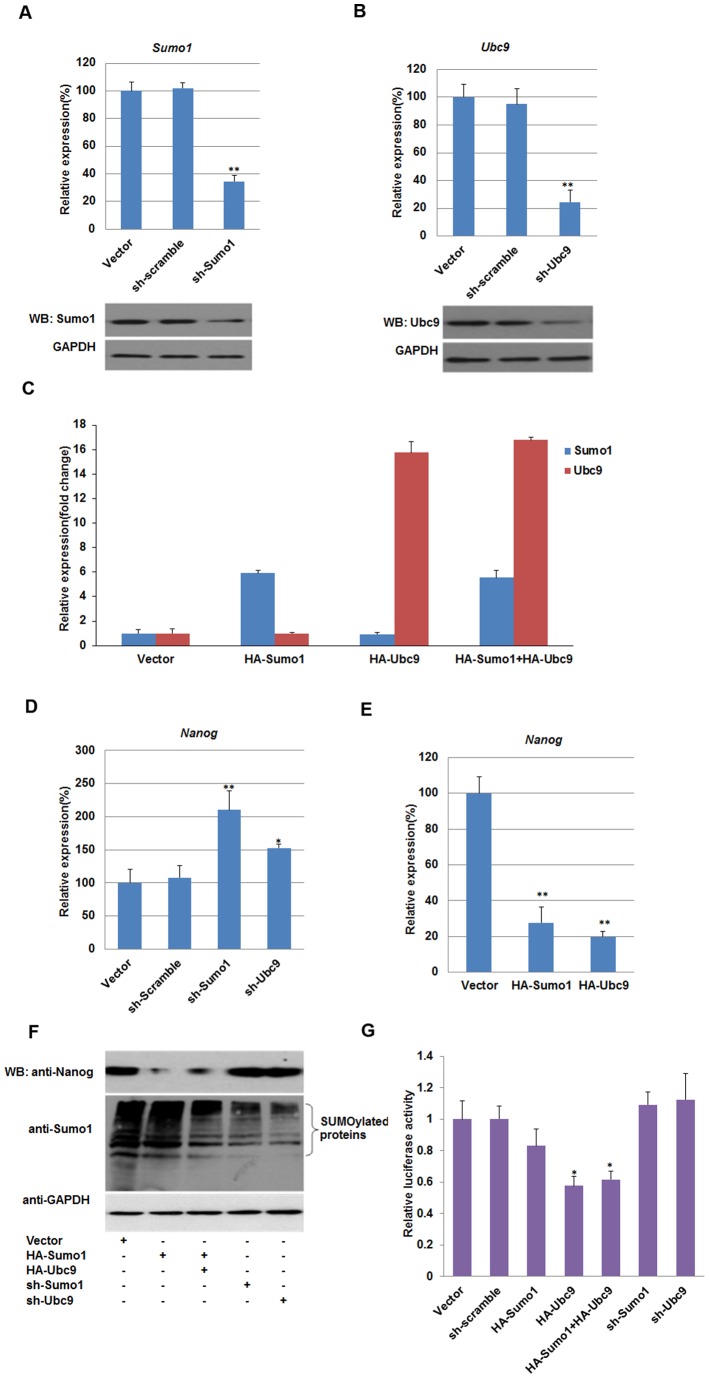
SUMOylation represses Nanog expression in F9 embryonal carcinoma cells. (A) Endogenous Sumo1 expression in control and Sumo1-knockdown F9 EC cells. After 48 hours post-transfection with a Sumo1-specific shRNA construct (sh-Sumo1) or negative control (Vector and sh-scramble), Sumo1 expression was determined by qPCR and western blot. (B) Endogenous Ubc9 expression in control and Ubc9-knockdown F9 EC cells. After 48 hours post-transfection with an Ubc9-specific shRNA construct (sh-Ubc9) or negative control (Vector and sh-scramble), Ubc9 expression was determined by qPCR and western blot. (C) Overexpression of Sumo1 and/or Ubc9 in F9 EC cells. F9 EC cells were transfected with pCMV-HA-Sumo1, pCMV-HA-Ubc9 and empty vector as indicated, Sumo1 and Ubc9 mRNA levels were detected by qPCR respectively. (D) qPCR analysis of Nanog expression in F9 EC cells in response to knockdown of Sumo1/Ubc9. (E) qPCR analysis of Nanog expression in F9 EC cells in response to overexpression of *Sumo1/Ubc9.* (F) Endogenous *Nanog* protein in F9 EC cells were determined by western blot after transient transfection with the indicated constructs. (G) Transcriptional activity of the *Nanog* proximal promoter in response to SUMOylation. After 48 hours post-transfection with the indicated plasmids, luciferase activity was determined and normalized against control empty vector transfection. qPCR data were normalized to *GAPDH.* Data are presented as the mean +/− SD and are derived from three independent experiments. *, p<0.05; **, p<0.01; WB: western blot.

SUMOylation is an important post-translational protein modification and regulates many critical cellular processes. SUMOylation of Sox2 inhibited its DNA binding activity and negatively regulated its transcriptional activity [15], while SUMOylation of Oct4 enhanced its stability, DNA binding, and transactivation [16], [17]. These data indicated that SUMOylation plays an important role in regulation of genes expression in ES cells. To gain a general understanding of the potential role of SUMO modification in ES cells, we reduced the SUMOylation level by knockdown of Sumo1/Ubc9, or increased the SUMOylation level by exogenously expressed Sumo1/Ubc9 in F9 embryonal carcinoma (F9 EC) cells. Real-time quantitative PCR (qPCR) and western blot results showed that short hairpin RNAi constructs could efficiently reduce the expression level of *Sumo1* and *Ubc9* compared with that of empty vector or scramble RNAi vector (Fig. 1A and B). Overexpression of Sumo1 and Ubc9 was detected in HA-*Sumo1* and HA-*Ubc9* transfected F9 EC cells (Fig. 1C). Under this condition, we measured the mRNA levels of key regulators *Nanog*, *Sox2* and *Oct4*, and found that *Nanog* transcripts were increased by 1.5–2-fold upon knockdown of Sumo1/Ubc9 (Fig. 1D). In contrast, overexpression of Sumo1/Ubc9 reduced the *Nanog* mRNA level to 20–30% compared with that of the control (Fig. 1E). The expression level of transcription factors *Sox2* and *Oct4* (*Pou5f1*) did not change significantly (data not shown). Consistent with qPCR results, overexpression of Sumo1/Ubc9 led to a dramatic reduction of Nanog protein compared with control cells, while knockdown of Sumo1/Ubc9 increased Nanog expression (Fig. 1F). To confirm these results, F9 EC cells were treated with ginkgolic acid, an inhibitor of SUMOylation for 10 h [19]. Then, we detected the SUMOylation level and Nanog expression by western blot and qPCR. The result further proved that Nanog gene expression is suppressed by SUMOylation (Fig. S1).

Dual-luciferase assays were performed to determine whether SUMOylation repressed the transcriptional activity of the *Nanog* proximal promoter. The proximal promoter sequence of *Nanog* containing the Sox2/Oct4 element (−230 to +50 relative to the transcription start site) was cloned and inserted into a pGL4.10 vector, then we cotransfected F9 EC cells with the reporter vector and shRNA or Sumo1/Ubc9 expression constructs. As shown in Figure 1G, low luciferase activity was observed in Sumo1/Ubc9-overexpressing cells. However, we noted that the luciferase activity of the Nanog proximal promoter did not increase significantly in Sumo1/Ubc9 knockdown cells (Fig. 1G), in F9 EC cells, there still be many other SUMO substrates except Oct4 and Sox2 (Fig. 1F), the global reduction of SUMOylation may decreases the SUMO modification of other protein, thereby disturbs the results. Taken together, these results suggested that SUMOylation suppresses Nanog expression *in vivo* through inhibiting the transcriptional activity of its proximal promoter.

### SUMOylation of Oct4 and Sox2 regulate *Nanog* by different ways

Next, we investigated how SUMOylation suppressed *Nanog* expression. Transcription factors Oct4 and Sox2 form a heterodimer and bind to the *Nanog* promoter [18]. Moreover, Oct4 and Sox2 have been shown to be modified by Sumo1 at Lysine 118 and lysine 247 respectively [15]. To test whether *Nanog* was regulated indirectly by SUMOylation of Oct4 and Sox2, we detected the expression patterns of Nanog in response to various levels of SUMOylated Oct4 and Sox2.

**Figure 2:**
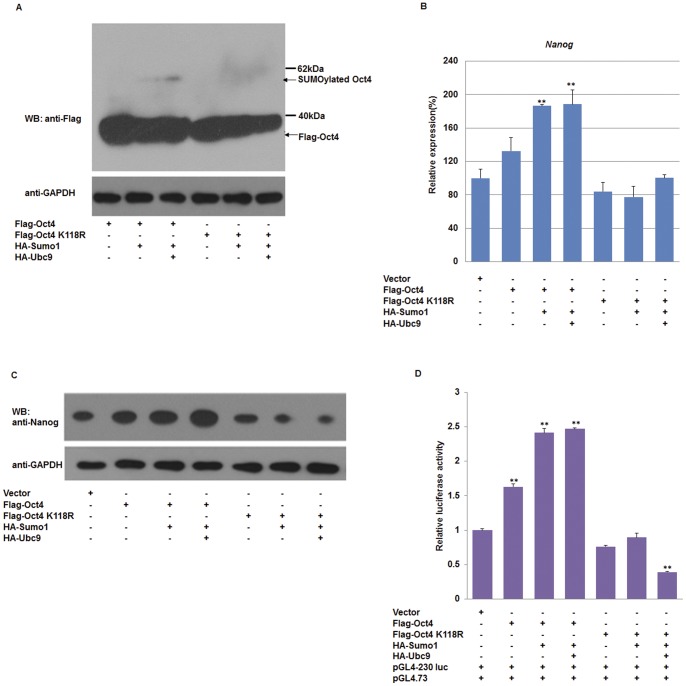
SUMOylation of Oct4 enhances Nanog expression. (A) Oct4 is modified by Sumo1 at Lysine 118. Wild-type Oct4 or the SUMO receptor site mutant Oct4 K118R was expressed in combination with HA-Sumo1 and HA-Ubc9 in F9 EC cells. (B) qPCR analysis of *Nanog* mRNA in response to various levels of SUMOylated Oct4. The levels of the transcripts were normalized against control empty vector transfection. (C) Western blot analysis of Nanog in F9 EC cells under a varying SUMOylation status of Oct4. (D) SUMOylation of Oct4 enhances the *Nanog* proximal promoter transcription. Transcriptional activities of the *Nanog* promoter (−230 to +50 bp relative to the transcription start site) in response to various levels of SUMOylated Oct4 were determined by dual-luciferase reporter assays. qPCR data were normalized to *GAPDH.* Data are presented as the mean +/− SD and are derived from three independent experiments. *, p<0.05; **, p<0.01; WB: western blot.

**Figure 3:**
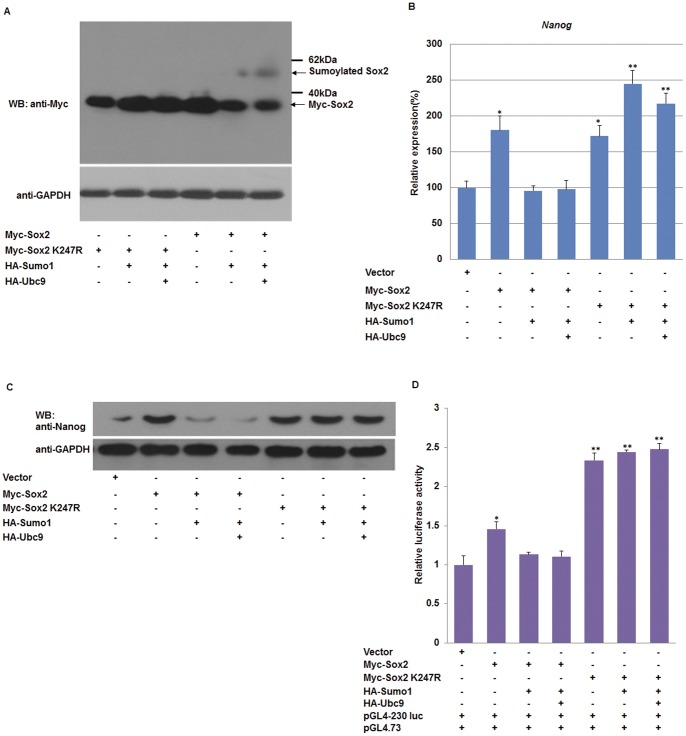
SUMOylation of Sox2 represses Nanog expression. (A) Covalent modification of Sox2 by Sumo1 at Lysine 247. Wild-type Sox2 and mutant Sox2 K247R were coexpressed with HA-Sumo1 and HA-Ubc9. (B) qPCR analysis of Nanog mRNA in response to various levels of SUMOylated Sox2. (C) Western blot analysis of Nanog in F9 EC cells under a varying status of SUMOylated Sox2. (D) Covalent modification of Sox2 with Sumo1 inhibits the transcriptional activity of the Nanog proximal promoter. Transcriptional activities of the Nanog proximal promoter (−230 to +50 bp relative to the transcription start site) in response to various levels of SUMOylated Sox2 were determined by dual-luciferase reporter assays. qPCR data were normalized to *GAPDH.* Data are presented as the mean +/− SD and are derived from three independent experiments. *, p<0.05; **, p<0.01; WB: western blot.

When co-overexpressed the Flag-Tagged Oct4 and HA-Tagged Sumo1, a high molecular mass band of a covalently modified form of Oct4 was detected and the band intensity increased with overexpression of Ubc9. In contrast, the modified band was rarely found when the SUMO acceptor site in Oct4 was mutated (Oct4 K118R) (Fig. 2A). SUMOylation of Sox2 and Sox2 K247R was also characterized. Compared with wild-type Sox2, the Sox2 K247R mutant failed to form higher molecular bands when it was co-transfected with HA-tagged Sumo1 or Ubc9 (Fig. 3A).

Subsequently, qPCR was used to quantify the *Nanog* transcripts in F9 EC cells expressing wild type Oct4 or Oct4 K118R. As shown in Figure 2, the relative transcription level of *Nanog* in cells co-expressing Oct4 and Sumo1 or Ubc9 was 1.5–2-fold higher than in Oct4 K118R-transfected cells. Consistent with qPCR results, the protein level of Nanog was increased by the SUMOylation of Oct4 in pluripotent cells (Fig. 2B and C). These results indicate that SUMOylation of Oct4 increases the expression of Nanog.

Furthermore, we examined the effect of SUMOylation of Sox2 on Nanog expression. As shown in Figure 3, co-overexpression of wild-type Sox2 and Sumo1/Ubc9 decreased the Nanog expression at both mRNA level and protein levels. Accordingly, compared with the control group, *Nanog* transcripts were increased by more than 1.5-fold in Sox2 K247R-transfected cells, in which Sox2 was not modified by Sumo1. The results of western blot confirmed that Nanog expression was inhibited by SUMOylated Sox2 (Fig. 3B and C). Luciferase assays showed that SUMOylation of Oct4 promoted Nanog transcription (Fig. 2D), while covalent modification of Sox2 with Sumo1 reduced Nanog transcription (Fig. 3D).

**Figure 4:**
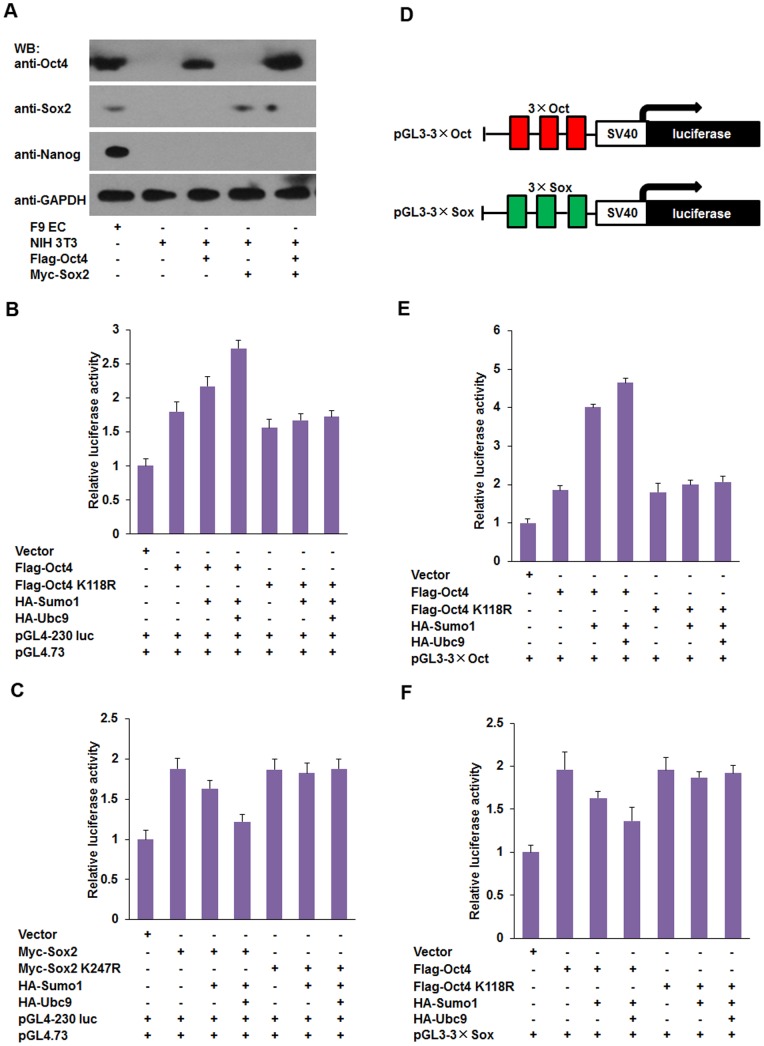
SUMOylation regulates transactivity of Oct4 and Sox2. (A) NIH3T3 cells lack the expression of pluripotency genes. Detection of Oct4, Sox2 and Nanog protein expression in wild-type and Flag-Oct4 or Myc-Sox2-transfected NIH3T3 cells, F9 EC cells lysate was used as a positive control. (B) SUMOylation of Oct4 enhances the *Nanog* proximal promoter transcription in NIH3T3 cells. NIH3T3 cells were transfected with the indicated plasmids, and dual luciferase assays were performed at 48 hours post-transfection. (C) SUMOylation inhibits the transcriptional activity of Sox2 in NIH3T3 cells. NIH3T3 cells were transfected with the indicated plasmids, and then dual luciferase assays were performed at 48 hours post-transfection. (D) Schematic representation of Octamer/Sox single site reporter constructs. The construct consisted of the firefly luciferase gene driven by the SV40 promoter and three tandem copies of the Octamer/Sox element. (E) SUMOylation of Oct4 promotes the transcriptional activity of pGL3-3×Oct. NIH3T3 cells were cotransfected with pGL3-3×Oct and various combinations of plasmids, and then luciferase activity was determined at 48 hours post-transfection. (F) SUMOylation of Sox2 decreases the transcriptional activity of pGL3-3×Sox. NIH3T3 cells were cotransfected with pGL3-3×Sox and various combinations of plasmids, and then luciferase activity was determined at 48 hours post-transfection. Data are presented as the mean +/− SD and are derived from three independent experiments. WB: western blot.

As shown in Figures 2 and 3, we noted that cotransfection of Oct4 K118R or Sox2 with Sumo1 and Ubc9 caused the expression level of Nanog to fall back to the baseline level. This effect may be due to the complexity of endogenous Oct4 and Sox2, because the Oct4/Sox2 dimers would be altered by manipulating either Oct4 or Sox2. To overcome this effect, we performed additional experiments using NIH 3T3 cells that do not express endogenous Oct4 or Sox2 (Fig. 4A). Consistent with results using F9 EC cells, we found that SUMOylation of Oct4 promoted transcription of the Nanog proximal promoter (Fig. 4B), and SUMOylation of Sox2 decreased its transactivity for the Nanog proximal promoter (Fig. 4C). However, the luciferase activity did not fall back to the levels of the negative control, indicating that the SUMOylation levels of both exogenous and endogenous Oct4/Sox2 was altered by overexpression of Sumo1 and Ubc9 in F9 EC cells.

To further verify our results, single site Octamer (Oct)/Sox reporter assays were performed with NIH 3T3 cells and a pGL3-promoter construct, in which three tandem repeats of the Oct/Sox element had been introduced (Fig. 4D). Cotransfection of the 3×Oct reporter construct with Flag-Oct4 led to a 2-fold increase in luciferase activity, and the luciferase activity increased to 4–4.5 fold by cotransfecting Sumo1 and Ubc9 plasmids. In contrast, the luciferase activity of Oct4 K118R-transfected NIH 3T3 did not increase significantly, even with cotransfection of Sumo1 and Ubc9 plasmids (Fig. 4E). As shown in Figure 4F, cotransfection of the 3×Sox reporter construct with Myc-Sox2 led to increased luciferase activity by more than 1.5-fold, while luciferase activity decreased significantly when Sumo1 and Ubc9 plasmids were cotransfected. The luciferase activity of Sox2 K247R-transfected NIH 3T3 cells did not change significantly (Fig. 4F). Taken together, our data shows that SUMOylation of Oct4 and Sox2 regulates the *Nanog* proximal promoter by distinct mechanisms in which SUMOylation of Oct4 promotes *Nanog* expression, while SUMOylation of Sox2 inhibits *Nanog* expression.

### SUMOylation of Oct4 and Sox2 does not change their subcellular localization

**Figure 5:**
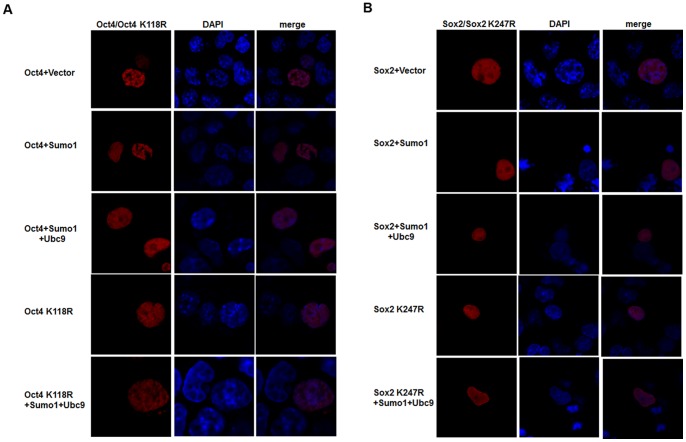
SUMOylation does not alter the subcellular localization of Oct4 and Sox2. (A) Subcellular localization of Oct4 and Oct4K118R. F9 EC cells were cotransfected with red fluorescent protein tagged Oct4/Oct4 K118R plasmids and HA-Sumo1 or HA-Ubc9. There is no obvious difference in the subcellular localization of Sumo1-modified and unmodified Oct4. (B) The distribution of Sox2 and Sox2 K247R in F9 EC cells. Cotransfection of F9 EC cells with pDsRed-*Sox2*/pDsRed-*Sox2* K247R and HA-Sumo1 or HA-Ubc9. Both SUMOylated Sox2 and unmodified Sox2 K247R localize in the nuclei. Nuclei were stained with DAPI (blue). Cells were observed and photographed under a Nikon confocal microscope at ×400 magnification.

It has been reported that SUMOylation modulates the function of some proteins, such as nucleophosmin/B23 and von Hippel-Lindau (VHL) tumor suppressor protein, by affecting their distribution between the cytoplasm and nuclei of mammalian cells [20], [21]. To investigate whether SUMOylation regulated Nanog expression by changing the subcellular localization of Sox2 and Oct4, red fluorescent protein (RFP)-tagged Oct4/Oct4 K118R and Sox2/Sox2 K247R were cotransfected with various combinations of plasmids into F9 EC cells. As shown in Figure 5, both SUMOylated Oct4/Sox2 and unmodified Oct4 K118R/Sox2 K247R were distributed in the nucleus, suggesting that SUMOylation of Oct4 and Sox2 did not change their subcellular localization. Moreover, we did not observe any obvious changes in the distribution of Oct4 and Sox2 within nucleus, by neither enhancing nor inhibiting SUMOylation (Fig. 5). These results are similar to those of a previous report and support that SUMOylation does not affect subcellular localization of Sox2 and Oct4 [16].

### SUMOylation disrupts the interaction between Sox2 and Oct4

**Figure 6:**
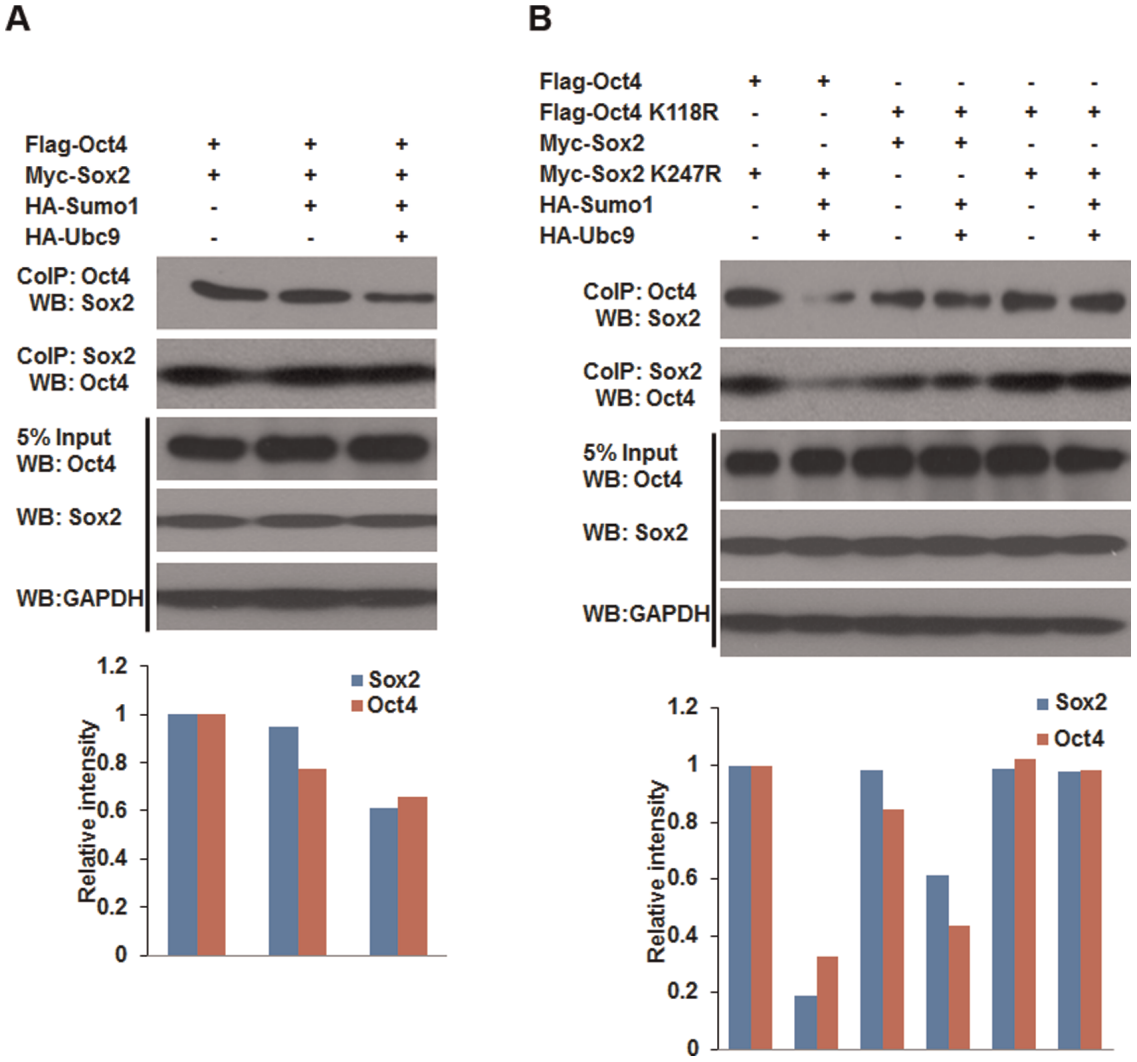
SUMOylation impairs the protein-protein interaction between Oct4 and Sox2. NIH3T3 cells were cotransfected with various combinations of Oct4/Oct4 K118R, Sox2/Sox2 K247R, HA-Sumo1 and HA-Ubc9 expression plasmids as indicated. Cell extracts were respectively co-immunoprecipitated with anti-Oct4 and anti-Sox2 antibody-coated affinity beads. Whole-cell lysates (input) and immunoprecipitated proteins were separated by 12% SDS-PAGE, followed by western blot with anti-Sox2, anti-Oct4, or anti-GAPDH antibodies. Western blot images were analyzed using Image J. (A) The protein-protein interaction between wild-type Oct4 and Sox2, the relative band intensity values of samples to controls were presented in bar histogram. (B) The protein-protein interaction between wild-type Oct4/Sox2 and mutant Sox2 K247R/Oct4 K118R, the relative band intensity values of samples to controls were presented in bar histogram. CoIP: co-immunoprecipitation; WB: western blot.

Protein-protein interactions are often regulated by posttranslational modifications, such as phosphorylation and SUMOylation. SUMOylation regulates protein-protein interactions by providing or masking protein-interacting surfaces. In ES cells, the heterodimer form of Oct4 and Sox2 is required to regulate other linage specific genes including *Nanog* [22], [23]. In order to test the effect of SUMOylation on the formation of Oct4-Sox2 heterodimer, co-immunoprecipitation (CoIP) experiment were performed using NIH 3T3 cells. As shown in Figure 6, the interaction between wild-type Sox2 and Oct4 was decreased when they were modified by Sumo1 compared to the interaction between unmodified Sox2 and Oct4, suggesting that SUMOylation impaired the binding affinity between Oct4 and Sox2. In addition, the suppressive effect of SUMOylation on Nanog expression via Oct4 and Sox2 may be partially due to the interference of heterodimer formation of Oct4/Sox2 by the modification of Sumo1.

### SUMO E3 ligase are involved in regulating Nanog expression

**Figure 7:**
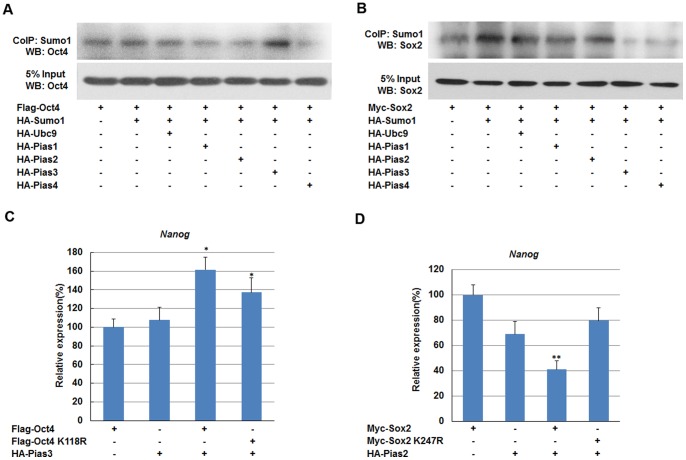
SUMO E3 ligases PIAS proteins mediate substrates-specific SUMOylation and regulate Nanog transcription. (A and B) Pias2 and Pias3 promote SUMOylation of Oct4 and Sox2, respectively. NIH3T3 cells were transfected with various combinations of plasmids as indicated. SUMOylated Oct4 and Sox2 was enriched by CoIP using an anti-Sumo1 antibody, and detected by western blot with anti-Oct4 and anti-Sox2 antibodies, respectively (upper panel). (C and D) *Nanog* transcription is up-regulated by Pias3, but down-regulated by Pias2. Transfection of F9 EC cells with various combinations of plasmids as indicated. The levels of *Nanog* transcripts were normalized against *GAPDH* expression. Data are presented as the mean +/− SD and are derived from three independent experiments. *: p<0.05; **: p<0.01. CoIP: co-immunoprecipitation; WB: western blot.

Furthermore, we tested the effect of SUMO E3 ligases on Nanog expression. After cotransfection of NIH3T3 cells with SUMO E3 ligases Pias1, Pias2, Pias3 and Pias4, and Oct4 or Sox2, cell lysates were precipitated using anti-Sumo1 antibody coated beads and analyzed by western blot. As shown in Figure 7, Pias3, and not other PIAS family E3 ligases, enhanced Oct4 SUMOylation as indicated by the high intensity of the SUMOylated Oct4 band was detected in sample cotransfected with Oct4 and Pias3 plasmids (Fig. 7A). However, Pias3 did not enhance the SUMOylation of Sox2. Instead, Pias2 was found to function as an E3 ligase toward Sox2 and enhanced its SUMOylation (Fig. 7B).

To explored the role of Pias2 and Pias3 in the *Nanog* transcription, we transfected Pias3 into F9 EC cells and found that Pias3 could induce the Nanog expression (Fig. 7C). As expected, we detected significantly reduced *Nanog* mRNA levels in the presence of Pias2 (Fig. 7D). Taken together, these data suggest that SUMO E3 ligases Pias2 and Pias3 suppress or induce Nanog expression by enhancing the SUMOylation of Sox2 or Oct4 respectively.

## Discussion

To our knowledge, this is the first study to investigate the regulation of SUMO on Nanog, a transcription factor required for maintaining the pluripotency of ES cells. We found that SUMOylation mediated a negative effect on *Nanog* expression, in which overexpression of the key components of the SUMO system decreased Nanog expression significantly. We also tried to elucidate the mechanisms of SUMOylation, which regulate Nanog. We revealed that SUMO modification of Sox2 and Oct4 alters their transcriptional activity and interaction. Furthermore, the results showed that SUMO E3 ligases Pias2 and Pias3 are involved in regulating Nanog by enhancing SUMOylation of Sox2 and Oct4, respectively. Taken together, our study indicates that SUMOylation regulates Nanog by affecting transcription factors Sox2 and Oct4.

SUMOylation is a post-translational modification involved in various cellular processes, such as nuclear-cytosolic transport, transcriptional regulation, apoptosis, protein stability and the DNA damage response [24]–[27]. Most recently, it has been reported that the SUMOylation pathway is involved in early development and the cellular pluripotency of vertebrates [28]–[31]. Additionally, some transcription factors, which function in the protein interaction network for the pluripotency of ES cells, are regulated by SUMOylation. These data suggests that SUMOylation has pivotal roles in cell differentiation and the maintenance of ES cell stemness.

Because the *Nanog* promoter has a Nanog consensus site upstream of the transcription start site [32], we considered that Sumo1 may covalently modify Nanog as a feedback response to repress *Nanog* expression. We analyzed the Nanog amino acid sequence using the SUMOsp 2.0 software [33], but no potential SUMOylation site Ψ-K-X-E (where Ψ represents a hydrophobic amino acid, and X represents any amino acid) was found. Next, we focused on two SUMO targets, Oct4 and Sox2, which positively regulate *Nanog* by binding to the proximal promoter of *Nanog*. However, our results showed that SUMOylation of Oct4 and Sox2 had opposing effects on Nanog expression. SUMOylated Oct4 enhanced Nanog expression (Fig. 2), and conversely, SUMOylated Sox2 downregulated Nanog expression (Fig. 3). Additional luciferase assays demonstrated that SUMOylation repressed *Nanog* transcription via modulating Oct4/Sox2 binding to the Oct/Sox element in the *Nanog* proximal promoter region (Fig. 2D, 3D and 4D). This effect might be due to the fact that SUMOylation diminishes the DNA binding activity of Sox2, but enhances Oct4 binding to DNA octamer element [15], [16]. F9 EC cells express endogenous Oct4 and Sox2, hence the dynamic of the endogenous Oct4/Sox2 dimers that bind to the *Nanog* promoter would be altered by adjusting the SUMOylation levels of exogenous Oct4/Sox2, thereby further disturbing the endogenous effect of SUMOylation on Nanog transcription. To reduce the complexity introduced by endogenous Oct4/Sox2, we performed reporter assays with NIH 3T3 cells, and the results further demonstrated that SUMOylation of Oct4 enhances its transactivation, and covalent modification of Sox2 with Sumo1 decreases the transactivation ability of the *Nanog* proximal promoter (Fig. 4).

In some cases, SUMOylation modulates target protein function by altering their subcellular or subnuclear localization [34]–[36]. In this study, to determine whether SUMOylation altered the subcellular localization of Sox2 and Oct4, we investigated the intracellular distribution of Sox2 and Oct4 in pluripotent F9 EC cells. The results showed that the subcellular localization of Sox2 and Oct4, which are normally localized in the nucleus, was not affected by SUMOylation. Therefore, our findings indicate that SUMOylation regulates the transcriptional activity of Sox2 and Oct4 by a mechanism other than altering nuclear localization.

It is well known that Oct4 and Sox2 are necessary to maintain the pluripotency of ES cells. In ES cells, Oct4 and Sox2 often form the Oct4-Sox2 heterodimer to regulate the expression of *Nanog* and other target genes, and an Oct4-centered transcriptional network controls the pluripotent cell identity [37], [38]. In our current study, the effect of SUMO modification on the protein-protein interaction between Oct4 and Sox2 was evaluated by CoIP and western blot. The results demonstrated that the Oct4-Sox2 interaction (dimerization) was impaired by SUMOylation, in which both SUMOylated Sox2 and Oct4 showed a reduced protein-protein binding ability. Accordingly, we speculated that the stability of Sox2 and Oct4 binding to the *Nanog* promoter may be impaired by SUMOylation.

E3 ligases contribute to SUMOylation substrate specificity and efficiency [11]. Three main subtypes of SUMO E3 ligases have been identified: Pias proteins, RanBP2, and Pc2 [12], [39], [40]. In the present study, we found that SUMO E3 ligase Pias2 promoted SUMOylation of Sox2 and repressed *Nanog* transcription, while Pias3 enhanced SUMO modification of Oct4 and enhanced its transactivation. Hence, we hypothesize that when Sumo1 and Ubc9 are overexpressed in F9 EC cells, the SUMOylation of endogenous Sox2 is enhanced by specific E3 ligases such as Pias2, while the amount of SUMOylated Oct4 is much less than that of Sox2 because of the specificity of E3 ligases. Under such circumstances, Nanog expression is mainly regulated by SUMOylated Sox2, thus resulting in a decreased amount of Nanog.

Nanog plays a crucial role in maintenance of the undifferentiated state of mouse ES cells, and downregulation of Nanog induces differentiation of human ES cells [41]. Although no obvious phenotype change was observed when we overexpressed Sumo1 and Ubc9 in F9 EC cells, based on our studies, we believe it is possible to induce ES cell differentiation into specific cell types by combining SUMOylation modification with small molecule treatments.

In conclusion, our findings demonstrate that SUMOylation represses Nanog expression. On the one hand, SUMOylation of Sox2 inhibits its transcriptional activity and represses *Nanog* transcription, while SUMOylation also disturbs the protein-protein interaction between Oct4 and Sox2, resulting in decreased of Nanog expression. Additionally, previous studies showed that there are several other SUMO substrates that express specifically in ES cells, such as SALL1 and Klf4, SUMOylation modulates their transcriptional activity, and these genes are involved in regulating Nanog expression [30], [31], [42], [43]. Therefore, we speculate that SUMOylation of pluripotency factors may be an alternative mechanism to control the Nanog level *in vivo*. Furthermore, SUMO E3 ligases might be a potential regulator involved in the regulation of Nanog expression, and further experiments will be needed to investigate the gene expression patterns and substrate-specificity of SUMO E3 ligases in undifferentiated and differentiated ES cells. Identification of the expression difference and target specificity of SUMO E3 ligases will be helpful to further understand the role of the SUMOylation pathway in cell-fate determination of pluripotent cells.

## Materials and Methods

### Reagents

Unless otherwise indicated, reagents were purchased from Sigma Chemical Co. (St. Louis, MO, USA). Primary antibodies mouse anti Flag, mouse anti Myc, mouse anti Oct4, goat anti-Sox2, mouse anti-Sumo1 and mouse anti-GAPDH were purchased from Santa Cruz Biotechnology Inc. (Santa Cruz, CA, USA). The Rabbit anti-Nanog polyclonal antibody was obtained from Bethyl Laboratories (Montgomery, TX, USA). Anti-rabbit, anti-mouse and anti-goat horseradish peroxidase (HRP)-conjugated secondary antibodies were obtained from the Beyotime institute of biotechnology (Jiangsu, China).

### Cell culture and transfection

All cell culture reagents were purchased from Gibco (Invitrogen, Carlsbad, CA, USA). Sterile plastic ware was purchased from Nunclon (Roskilde, Denmark). Mouse F9 embryonal carcinoma (EC) cells were purchased from the cell bank of the Chinese Academy of Sciences and maintained in 0.1% gelatin-coated plates with Dulbecco's modified Eagle's medium supplemented with 10% fetal bovine serum. NIH3T3 cells (CRL-1658, purchased from the American Type Culture Collection) were cultured in Dulbecco's modified Eagle's medium supplemented with 10% fetal bovine serum. All experimental cultures were incubated at 37°C in a moist atmosphere of 95% air and 5%CO_2_. Transfections were performed with FuGENE HD reagent (Roche, Basel, Switzerland) according to the manufacturer's instructions.

### Construction of plasmids

The pSilencer2.1-U6 hygro plasmid (Applied Biosystems, Foster City, CA, USA) was used for DNA vector-based shRNA construction. Sequences of shRNA for RNAi were as follows: *Sumo1* forward: GATCCGAATCATACTGTCAAAGACTTCAAGACGGTCTTTGACAGTATGATTCTTTTTTGTCGACA; *Sumo1* reverse: AGCTTGTCGACAAAAAAGAATC

ATACTGTCAAAGACCGTCTTGAAGTCTTTGACAGTATGATTCG; *Ubc9* forward: GATCCAAGCAGAGGCCTACACAATTTTTCAAGACGAAATTGTGTA

GGCCTCTGCTTTTTTTTGTCGACA; *Ubc9* reverse: AGCTTGTCGACAAAAA

AAAGCAGAGGCCTACACAATTTCGTCTTGAAAAATTGTGTAGGCCTCTGCTTG. Negative control sequences (scramble) were as follows: forward: GATCCGAAAGTAGAGCGCAGAACTTTCAAGACGAGTTCTGCGCTCTACTTTCTTTTTTGTCGACA; reverse: AGCTTGTCGACAAAAAAGAAAGTAGAGCGC

AGAACTCGTCTTGAAAGTTCTGCGCTCTACTTTCG. The *Ubc9* RNAi target sequence has been reported elsewhere [44]. These sequences were cloned into the pSilencer 2.1-U6 hygro plasmid in accordance with the manufacturer's instructions. The knockdown efficiency was examined by quantitative real time PCR (qPCR) and western blot.

Full-length cDNAs encoding mouse *Sumo1, Ubc9, Sox2, Pias1, Pias2, Pias3* and *Pias4* were obtained by RT-PCR of total RNA extracted from F9 embryonal carcinoma (EC) cells. The open reading frame (ORF) sequences of Sox2 with mutated SUMO accepter site (*Sox2* K247R) was produced by overlapping extension PCR, then *Sumo1, Ubc9, Pias1, Pias2, Pias3* and *Pias4* were inserted into a pCMV-HA plasmid. *Sox2 and Sox2* K247R were inserted into a pCMV-Myc plasmid. *Sox2 and Sox2* K247R were inserted into a pDsRed-N1 plasmid. pcDNA3-Flag-*Oct4* and pcDNA3-Flag-*Oct4* K118R plasmids were a kind gift from Michael L. Atchison (University of Pennsylvania, Philadelphia, PA, USA) [16]. *Oct4* and *Oct4* K118R were amplified by PCR using the pcDNA3-Flag-*Oct4* and pcDNA3-Flag-*Oct4* K118Rconstructs as templates and then inserted into a pDsRed-N1 plasmid.

The reporter plasmid pGL4-230 Luc reporter plasmid, containing the −230 to +50 region of the mouse *Nanog* promoter was constructed by a PCR-based method. To construct single site reporter plasmids, a synthetic oligonucleotide containing three tandem copies of the Oct and Sox elements (3×Oct: TTACAGCTTCTTTTGCATTCCATGTTACAGCTTCTTTTGCATTCCATGTTACAGCTTCTTTTGCATTCCATG; 3×Sox: TTACAGCTTCTACAATGTCCATGTTACAGCTTCTACAATGTCCATGTTACAGCTTCTACAATGTCCATG) were respectively cloned into a pGL3-promoter vector. All constructs were confirmed by DNA sequencing.

### Luciferase reporter assay

Luciferase measurements were performed with the Dual-Luciferase Reporter Assay System (Promega, Madison, WI, USA) according to the manufacturer's instructions. F9 EC cells were transfected with reporter constructs and various expression vectors with FuGENE HD following the manufacturer's protocol. The Renilla luciferase plasmid pGL4.73 was cotransfected as an internal control. After 48 hours post-transfection, cells were lysed with 200 μL/well (12 well plate) 1× passive lysis buffer for 15 minutes with shaking. 20 μL of each lysate was transferred to a 96 well plate and assayed by addition of 100 μL Luciferase Assay Reagent and 100 μL Stop & Glo Reagent. Data were collected with a VICTOR X5 Multilabel Plate Reader (PerkinElmer, USA).

### Reverse transcription PCR (RT-PCR) and quantitative real time PCR (qPCR)

Total RNA was isolated from F9 EC cells using Trizol reagent (Invitrogen, Carlsbad, CA, USA) according to the manufacturer's instructions. Purified RNA was reverse-transcribed using a SYBR PrimeScriptTM RT–PCR Kit (TaKaRa, Dalian, China). Real-time quantification of mouse *Sumo1, Ubc9, Nanog, Oct4, Sox2* mRNA was performed with an ABI StepOnePlus PCR system (Applied Biosystems, Foster City, CA, USA) using SYBR Premix ExTaq II (TaKaRa). The comparative Ct method was used to calculate the relative quantity of the target gene mRNA, normalized to Glyceraldehyde 3-phosphate dehydrogenase (*GAPDH*) and relative to the calibrator, and was expressed as the fold change  = 2−ΔΔCt [45]. The following conditions were used for qPCR experiments: 30 seconds at 95°C, followed by 40 cycles of 5 seconds at 95°C and 30 seconds at 60°C. Primer sequences used for qPCR have been described elsewhere [46], [47].

### Co-immunoprecipitation

NIH3T3 cells were transfected with various combinations of constructs as indicated. After 48 hours post-transfection, cells were collected and lysed in 1× IP buffer [50 mM Tris-HCl (pH 8.0), 150 mM NaCl, 1 mM EDTA, 1% Nonidet P-40, 10% glycerol, 50 mM *N*-ethylmaleimide and protease inhibitor cocktail (Roche)] on ice for 20 minutes. Protein extracts were incubated overnight with mouse anti-Oct4 or goat anti-Sox2 antibodies at 4°C overnight on a rotator. The next day Pierce protein A/G beads were added followed by incubation at 4°C for 3 hours. The beads were washed twice with 1× IP buffer, and then resuspended with 30 μL 1× SDS loading buffer and placed in a 95°C heat block for 5 min. The supernatant was then used for western blot assay.

### Western blot analysis

For western blot, 12% acrylamide gels were used. Separated proteins were transferred to PVDF membranes (Millipore, Bedford, MA, USA) for 2.5 h at 100 V, and the membranes were blocked in 5% non-fat milk powder/TBST for 2 hours. Then the membranes were incubated with the primary antibody at 4°C overnight. After being washed three times with TBST, the membranes were incubated further with secondary antibody for 2 h. After washing three times for 10 minutes each, immunoblots were revealed by autograph using SuperSignal west pico substrate (Pierce/Thermo Scientific, Rockford, IL, USA). The intensity of protein bands was quantified using Image J software.

### Determination of Oct4 and Sox2 Subcellular localization

Cotransfection of F9 EC cells using red fluorescent protein-tagged Oct4/Oct4 K118R, Sox2/Sox2 K247R with pCMV-HA-*Sumo1* or pCMV-HA-*Ubc9* plasmids. 48 hours after transfection, cells were washed three times in PBS and fixed with 4% paraformaldehyde in PBS for 15 minutes at room temperature, and permeablized with 0.1% Triton X-100 in PBS for 10 minutes. Then nuclei were stained with DAPI for 10 minutes. Cells were observed and photographed under a Nikon confocal microscope (Nikon, Tokyo, Japan).

### Statistical analysis

Data were reported as the mean ± standard deviation (SD), and analyzed using Student's t-test. p values<0.05 were considered significant.

## Supporting Information

Figure S1 **Effect of SUMOylation levels on Nanog expression in F9 embryonal carcinoma cells.** (A) Treatment with ginkgolic acid enhances *Nanog* transcription. F9 EC cells were treated with DMSO and 100 or 200 μM ginkgolic acid for 10 hours, and then qPCR was performed to examine the relative expression of *Nanog.* (B) Ginkgolic acid inhibits protein SUMOylation and promotes Nanog expression *in vivo*. F9 EC cells were treated with DMSO and 100 or 200 μM ginkgolic acid (100 μM or 200 μM) for 10 hours. Cells were lysed in RIPA buffer containing 50 mM N-ethylmaleimide, and then lysates were separated by 10% SDS-PAGE, followed by western blot with anti-Sumo1, anti-Nanog and anti-GAPDH antibodies respectively. Data are presented as the mean +/− SD and are derived from three independent experiments. *: p<0.05;**: p<0.01. WB: western blot. GA: ginkgolic acid.
(TIF)
