# Supplementary figures and images for: SUMOylation Represses Nanog Expression via Modulating Transcription Factors Oct4 and Sox2
Source: PLoS One. 2012 Jun 22;7(6):e39606. doi: 10.1371/journal.pone.0039606 (PMC3382131; doi:10.1371/journal.pone.0039606)

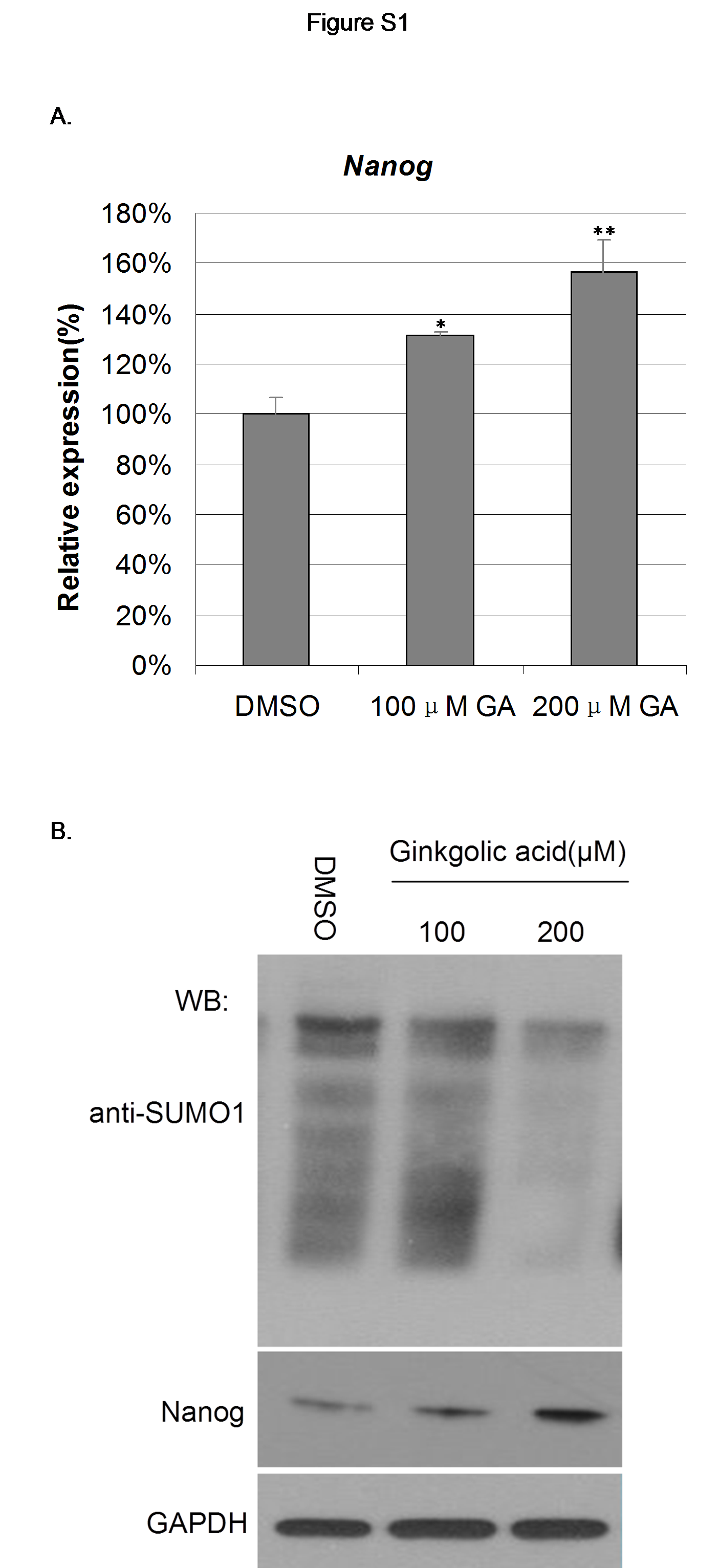

Supplement: Figure S1 — Effect of SUMOylation levels on Nanog expression in F9 embryonal carcinoma cells. (A) Treatment with ginkgolic acid enhances Nanog transcription. F9 EC cells were treated with DMSO and 100 or 200 μM ginkgolic acid for 10 hours, and then qPCR was performed to examine the relative expression of Nanog. (B) Ginkgolic acid inhibits protein SUMOylation and promotes Nanog expression in vivo. F9 EC cells were treated with DMSO and 100 or 200 μM ginkgolic acid (100 μM or 200 μM) for 10 hours. Cells were lysed in RIPA buffer containing 50 mM N-ethylmaleimide, and then lysates were separated by 10% SDS-PAGE, followed by western blot with anti-Sumo1, anti-Nanog and anti-GAPDH antibodies respectively. Data are presented as the mean +/− SD and are derived from three independent experiments. *: p<0.05;**: p<0.01. WB: western blot. GA: ginkgolic acid. (TIF) [file pone.0039606.s001.tif]
